# Evaluation of circulating vascular endothelial growth factor and its soluble receptors in patients suffering from persistent allergic rhinitis

**DOI:** 10.1186/s13223-016-0124-2

**Published:** 2016-04-27

**Authors:** E. Koczy-Baron, A. Grzanka, J. Jochem, R. Gawlik, A. Kasperska-Zajac

**Affiliations:** Department of Dermatology and Venerology, Bytom, Poland; Department of Internal Diseases, Dermatology and Allergology, SMDZ in Zabrze, Medical University of Silesia, Katowice, Poland; Departament of Basic Medical Sciences, Medical University of Silesia, Katowice, Poland; Clinical Department of Internal Diseases, Allergology and Clinical Immunology, SMDZ in Zabrze, Medical University of Silesia, Katowice, Poland

**Keywords:** Allergic rhinitis, Vascular endothelial growth factor, VEGF receptors, House dust mite, Platelet-poor plasma

## Abstract

**Background:**

Overexpression and enhanced release of vascular endothelial growth factor (VEGF) have been detected in various types of allergic inflammation, including asthma.

**Aim:**

To further evaluate the pattern of systemic release of VEGF in atopic allergy, free circulating VEGF was measured in patients with persistent allergic rhinitis (PAR).

**Methods:**

The concentrations of VEGF and its soluble receptors (sVEGF-R1 and VEGF-R2) in plasma were measured in patients with PAR sensitized to house dust mites and the healthy subjects.

**Results:**

No significant differences were found between PAR patients and healthy subjects with respect to plasma levels of VEGF and its receptors.

**Conclusions:**

It seems that free circulating VEGF may not be elevated in PAR patients. Moreover, on the basis of the present study as well as the earlier ones, it appears likely that systemic release of VEGF varies among patients with distinct clinical manifestation of atopy; may depend on severity/activity and the extent of inflammatory response.

## Background

It has been indicated that vascular endothelial growth factor (VEGF) is a multifunctional cytokine, which plays a role in the pathogenesis of immune-inflammatory reactions [1, 2]. VEGF is a powerful enhancer of vascular permeability and its activity is 50,000 times more potent than histamine [3]. In addition, VEGF may be released by different cells associated with allergic inflammation of the airways, including mast cells upon activation via the Fcε receptor I (FcεRI) [2, 4–6].

Interestingly, it has been demonstrated that dust-mite allergen may stimulate airway cells to increased VEGF secretion [7], which may play an important role in the modulation of eosinophilic inflammation [8]. There have been several reports describing association between VEGF and allergic airway inflammation. However, data regarding a role of VEGF in the upper-airway allergic diseases are scarce. In the present study, we investigated plasma VEGF concentration in patients with allergy to house dust mites, suffering from persistent allergic rhinitis (PAR) in the absence of asthma symptoms.

## Methods

Sixteen patients with moderate to severe PAR (untreated, newly diagnosed) without any history of asthmatic symptoms (seven males, nine females; aged 18–36 years; median 23 years) were enrolled into the study.

They had positive skin tests to *Dermatophagoides pteronyssinus* and *Dermatophagoides farinae* extract and positive serology (specific IgE = class 2 or higher) as well as negative response to all other common aeroallergens: pollens (grasses, cereals, trees and weeds), moulds (Alternaria, Cladosporium, Aspergillus, Penicillium), animal danders (comprising dog, cat, rabbit and guinea pig).

The clinical diagnosis of allergic rhinitis was performed according to criteria reported in the ARIA document.

The patients were compared with 35 healthy non-atopic subjects (20 males, 15 females; aged 18–38 years; median 21 years). None of the subjects had any other concomitant disorders [9].

All the subjects submitted respective written consent and the study was approved by the University Committee of Ethics.

### Blood samples and analytical methods

#### VEGF analysis

Because platelets are potential source of VEGF, we measured VEGF concentration in platelet-poor plasma (PPP) according to method described previously [9].

VEGF concentrations were determined using the enzyme-linked immunosorbent assay (ELISA) (Quantikine R&D Systems Inc. Minneapolis, MN USA). The detection limits were 9.0 pg/ml. Values below 9 pg/ml were equalized to zero.

#### sVEGF-R1 and sVEGF-R2 analyses

sVEGF-R1 and sVEGF-R2 concentrations were performed in the plasma collected, using EDTA as an anticoagulant. The cytokines concentrations were assayed by specific, commercially available, ELISA assay kits (Quantikine R & D Systems Inc., Minneapolis, MN USA) in accordance with the manufacturer’s instructions. The sensitivity of the assay for VEGF-R1 and sVEGFR-2 was 3.0 and 5 pg/ml, respectively.

#### Skin prick tests

Allergic status was evaluated using a panel of common inhalant (Allergopharma, Reinbeck, Germany). The skin wheal-flare reaction was read after 15 min and considered positive if the wheal diameter was at least 3 mm larger than one formed by the control substance.

#### Other laboratory investigations

The specific IgE to *D. farinae* and *D. pteronyssinus* were measured by ELISA using a commercial kit (Allergopharma, Reinbeck, Germany) according to the manufacturers’ instructions.

### Statistical analysis

Data were delivered as medians and interquartile range (IQR). All the statistical evaluations were performed by Mann–Whitney *U* test. The correlations between parameters were measured with Spearman rank test. The results were considered significant when *P* < 0.05.

## Results

**Fig. 1:**
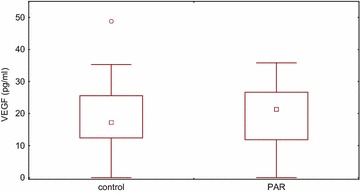
Plasma VEGF concentration in the control group and patients with persistent allergic rhinitis *PAR*

No significant differences were found between PAR patients and healthy subjects with respect to plasma levels of VEGF and its receptors (Table 1; Fig. 1). There were no significant correlations between values of VEGF and specific IgE to *D. farinae* and *D. pteronyssinus* (r = 0.244 and r = 0.165, p > 0.05, respectively).

**Table 1:** Plasma concentration of VEGF and its receptors in patients with PAR and healthy subjects

Parameters (units)	PAR (n = 16) *median* (IQR)	Healthy subjects (n = 35) *median* (IQR)	Significance
VEGF (pg/ml)	21.3 (0.0–35.8)	17.2 (0.0–48.8)	p > 0.05
sVEGF-R1 (pg/ml)	40.8 (26.4–70.2)	35.8 (21.7–60.3)	p > 0.05
sVEGF-R2 (pg/ml)	7247.5 (4780–14,960)	7470 (3740–12,765)	p > 0.05

*n* number; *PAR* persistent allergic rhinitis; *IQR* interquartile range

## Discussion

Atopic diseases like asthma, rhinitis, and atopic dermatitis (AD) are characterised by local changes at the site of allergic processes, as well as systemic immune-inflammatory response. Similarities should be noted between various atopic diseases, but there are also differences, regarding the profiles of mediators released into the systemic circulation [10].

So far, it has been reported that VEGF levels are increased in induced sputum and plasma/serum of asthmatics; its overproduction may be implicated in chronic inflammation, asthma exacerbation, and the airway remodeling [11, 12]. In addition, it has been reported that patients with AD may show significantly increased concentration of VEGF in PPP [9]. It has been demonstrated that the vascular permeability of nasal mucosa was increased by VEGF [13] and this cytokine level in nasal discharge was significantly higher in patients with allergic rhinitis sensitized by house dust mites and pollens than in nonallergic rhinosinusitis [14, 15]. In addition, VEGF serum concentrations were significantly lower in asymptomatic pollen-dependent AR patients studied outside the pollen season than in normal individuals. Interestingly, the VEGF concentrations increased after sublingual immunotherapy and were similar to values of normal subjects [16, 17]. We did not find any significant differences in plasma levels of VEGF and its receptors between PAR patients sensitized to house dust mites and the healthy subjects, suggesting that free circulating VEGF may not be elevated in the persistent allergy. On the basis of our own results as well as those reported by Ciprandi et al. [16, 17], it may be suggested that permanent exposure to allergens, such as AR—induced by persistent allergy (due to mites, mould or danders) or related to allergen immunotherapy are associated with normal VEGF concentration. However, confirmation of such hypothesis would demand evaluation of VEGF in symptomatic patients with seasonal AR induced by pollens during the pollen season.

These findings, in conjunction with earlier data, indicate that differences may exist in systemic VEGF release between patients with distinct clinical manifestation of atopy.

Different cells involved in the pathogenesis of allergic inflammation are able to synthesize and release VEGF. Among them important storage site is formed by the platelets, which release VEGF upon activation in vivo. The measurement of VEGF concentration in PPP is more reliable to assess in vivo free circulating VEGF, which is released from platelets and white blood cells during clotting formation [18].

It has been reported that platelet activation measured by plasma concentration of platelet factor 4 and beta-thromboglobulin is increased in AD patients, asthma, but not in allergic rhinitis and urticaria [10, 19–22]. Because platelets are important sources of VEGF in the circulation, these findings have confirmed our earlier observations showing that enhanced platelet release reaction is not a phenomenon accompanying chronic allergic rhinitis.

## Conclusions

Contrary to the previous studies indicating that VEGF level was increased in plasma of allergic patients suffering from asthma and atopic dermatitis, it seems that free circulating VEGF may not be elevated in PAR patients.

Moreover, on the basis of the present study as well as the earlier ones, it appears likely that systemic release of VEGF varies among atopic patients and may depend first of all on severity/activity and the extent of inflammatory response.

## Abbreviations

AD: atopic dermatitis
ELISA: enzyme-linked immunosorbent assay
Fcε receptor I: FcεRI
PPP: platelet-poor plasma
PAR: persistent allergic rhinitis
VEGF: vascular endothelial growth factor

## References

[1] Takahashi H, Shibuya M (2005). The vascular endothelial growth factor (VEGF)/VEGF receptor system and its role under physiological and pathological conditions. Clin Sci (Lond).

[2] Lee CG, Link H, Baluk P, Homer RJ, Chapoval S, Bhandari V, Kang MJ, Cohn L, Kim YK, McDonald DM, Elias JA (2004). Vascular endothelial growth factor (VEGF) induces remodeling and enhances TH2-mediated sensitization and inflammation in the lung. Nat Med.

[3] Senger DR, Connolly DT, Van de Water L, Feder J, Dvorak HF (1990). Purification and NH2-terminal amino acid sequence of guinea pig tumor-secreted vascular permeability factor. Cancer Res.

[4] Gruber BL, Marchese MJ, Kew R (1995). Angiogenic factors stimulate mast-cell migration. Blood.

[5] Horiuchi T, Weller PF (1997). Expression of vascular endothelial growth factor by human eosinophils: upregulation by granulocyte macrophage colony-stimulating factor and interleukin-5. Am J Respir Cell Mol Biol.

[6] Boesiger J, Tsai M, Maurer M, Yamaguchi M, Brown LF, Claffey KP, Dvorak HF, Galli SJ (1998). Mast cells can secrete vascular permeability factor/vascular endothelial cell growth factor and exhibit enhanced release after immunoglobulin E-dependent upregulation of fc epsilon receptor I expression. J Exp Med.

[7] Capetandes A, Zhuang M, Haque FN, Xie L, Frieri M (2007). Vascular endothelial growth factor is increased by human pulmonary cells stimulated with Dermatophagoides sp. extract. Allergy Asthma Proc.

[8] Feistritzer C, Kaneider NC, Sturn DH, Mosheimer BA, Kähler CM, Wiedermann CJ (2004). Expression and function of the vascular endothelial growth factor receptor FLT-1 in human eosinophils. Am J Respir Cell Mol Biol.

[9] Koczy-Baron E, Jochem J, Kasperska-Zajac A (2012). Increased plasma concentration of vascular endothelial growth factor in patients with atopic dermatitis and its relation to disease severity and platelet activation. Inflamm Res.

[10] Kasperska-Zajac A, Rogala B (2007). Platelet activation during allergic inflammation. Inflammation.

[11] Asai K, Kanazawa H, Kamoi H, Shiraishi S, Hirata K, Yoshikawa J (2003). Increased levels of vascular endothelial growth factor in induced sputum in asthmatic patients. Clin Exp Allergy.

[12] Lee KY, Lee KS, Park SJ, Kim SR, Min KH, Choe YH, Lee YC (2008). Clinical significance of plasma and serum vascular endothelial growth factor in asthma. J Asthma.

[13] Matsune S, Ohori J, Yoshifuku K, Kurono Y (2010). Effect of vascular endothelial growth factor on nasal vascular permeability. Laryngoscope.

[14] Matsune S, Ohori J, Sun D, Yoshifuku K, Fukuiwa T, Kurono Y (2008). Vascular endothelial growth factor produced in nasal glands of perennial allergic rhinitis. Am J Rhinol.

[15] Benson M, Carlsson B, Carlsson LM, Wennergren G, Cardell LO (2002). Increased expression of vascular endothelial growth factor-A in seasonal allergic rhinitis. Cytokine.

[16] Ciprandi G, Murdaca G, Colombo BM, De Amici M, Marseglia GL (2008). Serum vascular endothelial growth factor in allergic rhinitis and systemic lupus erythematosus. Hum Immunol.

[17] Ciprandi G, Colombo BM, Murdaca G, De Amici M (2008). Serum vascular endothelial growth factor and sublingual immunotherapy. Allergy.

[18] Wynendaele W, Derua R, Hoylaerts MF, Pawinski A, Waelkens E, de Bruijn EA, Paridaens R, Merlevede W, van Oosterom AT (1999). Vascular endothelial growth factor measured in platelet poor plasma allows optimal separation between cancer patients and volunteers: a key to study an angiogenic marker in vivo?. Ann Oncol.

[19] Kasperska-Zajac A, Brzoza Z, Rogala B (2008). Seasonal changes in platelet activity in patients with pollen-induced seasonal allergic rhinitis and seasonal asthma. J Asthma.

[20] Kasperska-Zajac A, Rogala B (2005). Markers of platelet activation in plasma of patients suffering from persistent allergic rhinitis with or without asthma symptoms. Clin Exp Allergy.

[21] Kasperska-Zajac A, Rogala B, Nowakowski M (2005). Assessment of platelet activity, expressed by plasma levels of platelet factor 4 and beta-thromboglobulin in patients with chronic idiopathic urticaria. Exp Dermatol.

[22] Kasperska-Zajac A, Rogala B (2006). Platelet function in anaphylaxis. J Invest Allergol Clin Immunol.

